# Reduced plasma levels of neuregulin-1 are associated with adverse outcomes in patients with atherosclerotic cardiovascular disease

**DOI:** 10.3389/fcvm.2025.1600480

**Published:** 2025-07-17

**Authors:** Jie Liu, Huiyi Liu, Hui Geng, Yan Fan, Meilin Liu

**Affiliations:** Department of Geriatrics, Peking University First Hospital, Beijing, China

**Keywords:** neuregulin-1, ASCVD, atherosclerosis, MACCEs, prognosis

## Abstract

**Background:**

Neuregulin-1 (NRG-1), a stress-mediated paracrine transmembrane growth factor, plays vital roles in the pathophysiology of atherosclerosis, myocardial infarction, ischemia-reperfusion, heart failure (HF), cardiomyopathy and other cardiovascular diseases. This study aimed to assess plasma NRG-1 levels in atherosclerotic cardiovascular disease (ASCVD) patients and explore the relationship between NRG-1 levels and patient outcomes.

**Methods:**

Plasma NRG-1, monocyte chemotactic protein-1 (MCP-1), myeloperoxidase (MPO) and vascular cellular adhesion molecule-1 (VCAM-1) concentrations were quantified in 185 ASCVD patients and 185 age- and sex-matched controls. All ASCVD patients were followed up for 14–16 months, and major adverse cardiovascular and cerebrovascular events (MACCEs), including angina pectoris, nonfatal myocardial infarction, nonfatal stroke, new HF symptoms, and CVD-related death, were recorded.

**Results:**

ASCVD patients presented notably lower NRG-1 levels (123.45 ± 0.87 pg/ml, vs. 139.76 ± 0.83 pg/ml for controls, *P* < 0.001) and higher MCP-1, MPO and VCAM-1 levels. Circulating NRG-1 levels were negatively associated with MCP-1 (−0.278, *P* < 0.001), MPO (−0.171, *P* = 0.001) `and VCAM-1 (−0.351, *P* < 0.001) levels. Logistic regression analysis revealed that a high NRG-1 level was a significant protective effect against ASCVD (OR = 0.859, 95% CI = 0.821–0.900; *P* < 0.001). In the mediation analysis, MCP-1, MPO, and VCAM-1 explained 20.2%, 8.8%, and 30.1%, respectively, of NRG-1's association with ASCVD. After an average follow-up of 13.8 ± 1.7 months, the mean NRG-1 level was lower in patients with MACCEs than in patients without MACCEs (112.04 ± 1.24 pg/ml vs. 125.93 ± 0.90 pg/ml, *P* < 0.001). Kaplan-Meier survival analysis revealed that patients with plasma NRG-1 concentration <122.5 pg/ml had a lower survival rate than those with higher levels (*P* < 0.001). According to the adjusted models, NRG-1 was independently associated with a decreased risk of MACCEs [adjusted HR 0.857 (95% CI 0.809–0.908), *P* < 0.001].

**Conclusions:**

Reduced NRG-1 levels in ASCVD patients increased the risk of MACCEs. NRG-1 levels may serve as useful laboratory markers of ASCVD prognosis.

## 1 Introduction

Atherosclerotic cardiovascular disease (ASCVD) remains the main cause of mortality worldwide, accounting for approximately 17.6 million deaths annually (1). The primary pathological basis of ASCVD is atherosclerosis, which involves the activation of proinflammatory signaling pathways, the expression of cytokines/chemokines and increased oxidative stress (2). In stable ASCVD patients, many, but not all, patients have increased levels of markers of vascular inflammation, which contributes to atherosclerotic progression (3, 4). Over time, those plaques obstructing coronary arteries can become vulnerable due to persistent inflammatory activation, leading to plaque weakening and subsequent rupture, which results in acute myocardial ischemia and infarction (5). Moreover, the clinical consequences of atherosclerosis are most likely determined by the inflammatory environment, which is the result of a balance between proinflammatory and proresolving factors (6). The plasma concentrations of several inflammatory biomarkers, such as C-reactive protein (CRP) (7, 8), myeloperoxidase (MPO) (9), vascular cellular adhesion molecule-1 (VCAM-1) (10) and monocyte chemotactic protein-1 (MCP-1) (11), have been studied in recent years to evaluate their relationships with different CVD types since they may help achieve an optimal diagnosis or predict prognosis.

Recent studies of cardiac development have indicated that neuregulin-1 (NRG-1) has the potential to be used as a therapeutic tool in stem cell therapies, tissue engineering applications, and clinical diagnostics and treatment (12). NRG-1 belongs to the epidermal growth factor (EGF) family and acts as a stress-mediated paracrine transmembrane growth regulator, mainly originating from endocardial and microvasculature endothelial cells in the heart (13, 14). NRG-1 binds to the receptor tyrosine kinase erythroblastic leukemia viral oncogene homolog (ErbB), leading to homodimerization (ErbB4/4) or heterodimerization (ErbB2/3 or ErbB2/4) (15, 16). Upon ligand binding, activated NRG-1/ErbB can mediate the P13K/AKT and Erk/MAPK signaling pathways involved in several processes in cardiomyocytes (15, 17). Recent studies have shown that NRG-1 plays an important role in the occurrence and development of CVDs, such as heart failure (HF), myocardial infarction (MI)/ischemia‒reperfusion (IR), atherosclerosis, cardiotoxicity and arrhythmia. In particular, the cardioprotective effects of NRG-1 have been investigated broadly in HF patients. The molecular mechanisms by which NRG-1 therapy attenuates the progression of HF include preventing myocardial fibrosis and apoptosis (18), reducing oxidant-producing enzymes (19), and improving myocardial contractility and diastolic capacity (20, 21). Subsequent clinical trials also demonstrated promising results. Two phase II clinical trials reported that administering recombinant NRG-1 (rNRG-1) through daily intravenous infusions was safe and well tolerated in patients with stable chronic HF (22, 23). To examine the effects of rNRG-1 on overall survival, a phase III clinical trial is ongoing in a cohort of 1,600 patients with systolic HF receiving intravenous infusion of rNRG-1 (NCT03388593). Animal experiments demonstrated that rhNRG-1 significantly reduced the transverse plaque area in the arteria brachiocephalica of apoE/STZ mice (24).

However, NRG-1 levels in patients with ASCVD have not yet been reported, and the potential association between NRG-1 concentrations and clinical prognosis remains to be systematically investigated. The objective of the present study was to evaluate plasma NRG-1 levels in patients with ASCVD and explore their relationship with patient outcomes.

## 2 Materials and methods

### 2.1 Study design and population

The study was a prospective cohort analysis of consecutive subjects who were admitted to our hospital from March 2023 to December 2023. Eligible participants had a documented history of at least one risk factor for atherosclerosis, which included hypertension, hyperlipidemia, diabetes, current smoking, obesity [body mass index (BMI) ≥28 kg/m^2^] or a family history of early-onset ischemic heart disease. All patients who underwent carotid artery and peripheral vascular ultrasound, coronary computed tomography angiography (CTA) or CT angiography examination were divided into two groups: the ASCVD group, which included patients with ASCVD, and the control group, which included patients without ASCVD. The specific diagnostic criteria for ASCVD (25) include the following: (i) coronary atherosclerotic heart disease (CAD), in which the level of stenosis in at least one coronary artery, as verified by CTA or coronary angiography (CAG), is 50% or greater; (ii) cerebrovascular disease, in which carotid and/or intracranial artery stenosis of 50% or more is verified through vascular ultrasound, head and neck CT angiography, or angiography (26); and (iii) peripheral blood vessel blockage of 50% or more, as determined by CT angiography or peripheral vascular ultrasound.

The exclusion criteria for the current study were as follows: severe liver or renal organ dysfunction, death during hospitalization, end-stage malignant tumors, connective tissue disorders, severe acute infections, and patients receiving anti-inflammatory or immunosuppressive medications.

The research was carried out following the guidelines of the Declaration of Helsinki and received approval from the Ethics Committee of the Peking University First Hospital (approval number 2023-132-002).

### 2.2 Plasma sample collection and measurement of NGR-1 using enzyme-linked immunosorbent assay (ELISA)

The morning after admission, fasting venous blood samples were collected in tubes containing disodium ethylenediaminetetraacetic tetraacetic acid (1 mg/ml) and aprotinin (500 KIU/ml; Sigma, St. Louis, MO, USA) and then promptly centrifuged at 3,500 rpm for 10 min at 4°C. The resulting plasma was divided into several aliquots and stored at −80°C until it was analyzed in the laboratory. The plasma levels of NRG-1 were measured using a specific and quantitative ELISA kit (Jiangsu Meibiao Biotechnology Co., Ltd.) according to the manufacturer's protocol.

Briefly, 50 μl of standards or prediluted samples (1:4 in sample diluent) were added to the appropriate wells, which had been precoatd with an anti-NRG-1 mimonoclonal antibody. After incubation at 37°C for 30 min, add 50 μl biotinylated antibody to each well. After incubation at 37°C for 30 min, the plates were washed by wash buffer. Add 50 μl of substrate A followed by 50 μl of substrate B to each well, incubate at 37°C in the dark for 10 min, and then add 50 μl stop solution. Finally, the plates were read at 450 nm using a microplate reader. The detection range was from 3 to 150 pg/ml. All plasma samples were assayed in duplicate and mean values were calculated.

### 2.3 Data collection and follow-up

Demographic and clinical parameters were collected for all of the subjects at baseline. All participants were asked about their smoking status, family history of disease, use of medications, and medical history. Clinical parameters, including age, sex, BMI, blood pressure, heart rate (HR), and left ventricular ejection fraction (LVEF), were collected from medical records. Laboratory data, including liver and kidney function, uric acid, blood lipid, fasting blood glucose, and glycosylated hemoglobin data, were collected from the laboratory. The follow-up information for ASCVD patients was gathered by reviewing our medical records or by contacting the patients or their family members via phone every three months with a period of 14–16 months. The primary endpoint was the occurrence of major adverse cardiovascular and cerebrovascular events (MACCEs), including angina pectoris, nonfatal MI, nonfatal stroke, new HF symptoms, and CVD-related death. If the patient died, the cause of death was determined by taking a history from family members and reviewing medical records.

### 2.4 Statistical analysis

All the statistical analyses were performed using SPSS Statistics 26.0 software and R software version 4.3.1. Continuous variables are expressed as the mean ± standard deviation (SD) or error (SE), and categorical variables are presented as frequencies (percentages). Normally distributed quantitative data were analyzed via independent-samples two-tailed Student'*t*-tests. For nonparametric data, the Wilcoxon matched-pair signed-rank (two samples) test was used. Qualitative data were analyzed using the chi-square test (Fisher's exact test). Pearson/Spearman correlations and regression analyses were applied to assess the relation between NGR-1 and the inflammatory mediators. Binary logistic regression was used to determine whether NRG-1 was an independent predictor of ASCVD risk, considering various relevant covariates. These included the baseline characteristics of all participants (age, sex, BMI, and medical history) as well as risk factors for atherosclerosis [such as HbA1c, blood lipids, fasting blood glucose, creatinine (Cr), serum uric acid, and some inflammatory factors]. Mediation analysis was performed using R, along with Zstats v1.0 (https://www.zstats.net) to evaluate the proportional contributions of inflammatory factors (MCP-1, MPO, VCAM-1, and hs-CRP) to the associations of the NRG-1 with ASCVD risk.

Cardiovascular and cerebrovascular event rates were determined using the Kaplan‒Meier method and compared via the log-rank test. To determine the associations between baseline NRG-1 levels and the risk of MACCEs, cox proportional hazards models were used. Furthermore, to identify potential related changes, a variety of subgroup analyses and interaction analyses were performed. Patients with ASCVD were stratified into diverse subgroups, according to age (<60 vs. ≥60 years), sex, smoking status, and the presence of hypertension, dyslipidemia, diabetes, and hyperuricemia. A two-tailed *P* < 0.05 was considered to indicate statistical significance.

## 3 Results

### 3.1 Baseline characteristics of the study patients

**Figure 1:**
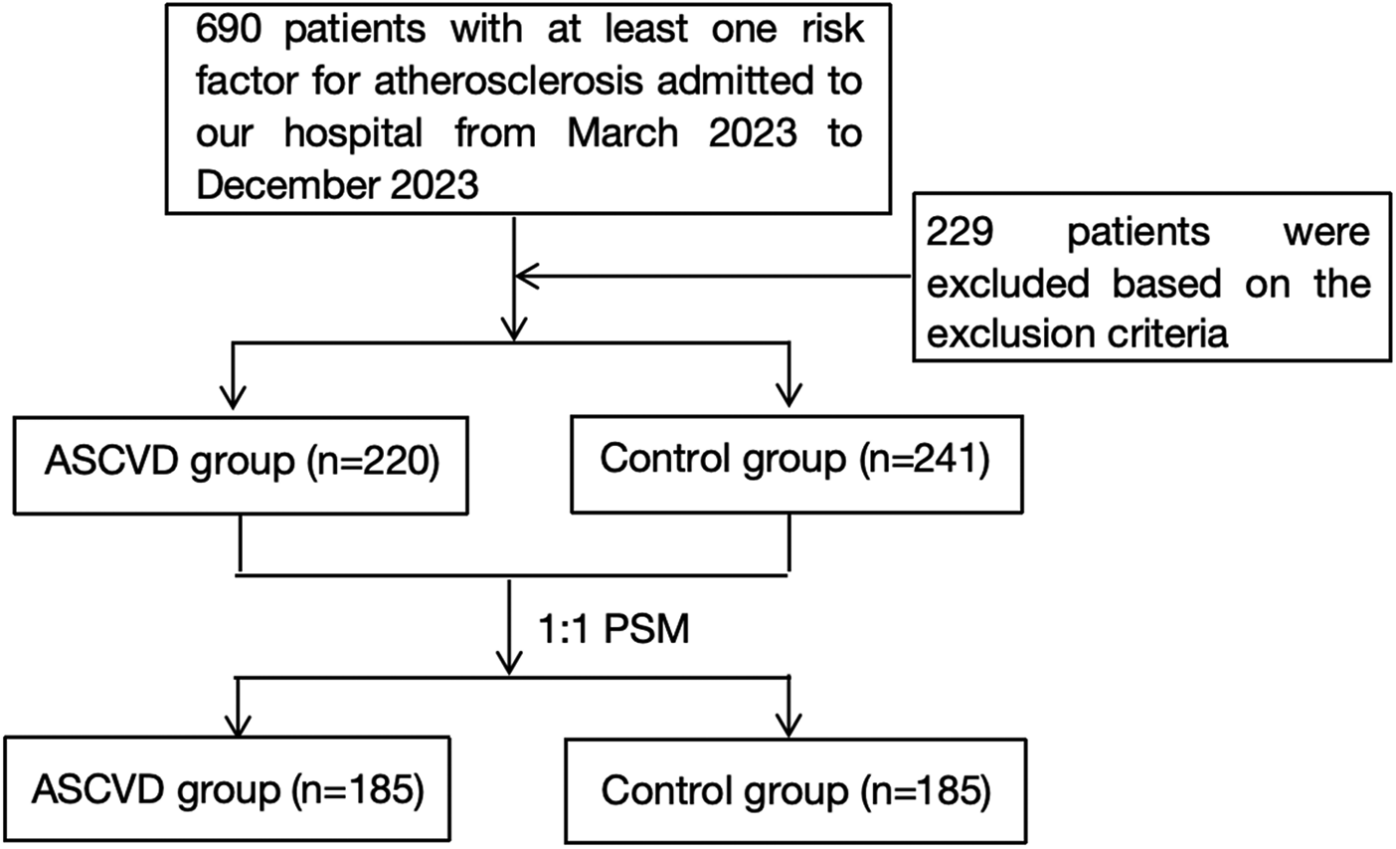
Flow diagram of the patient selection process.

A total of 690 subjects had at least one risk factor for atherosclerosis and were hospitalized during the study period; 229 were excluded based on the basis of the exclusion criteria (Figure 1). According to the inclusion and exclusion criteria, a total of 461 patients were selected and divided into the ASCVD group (*n* = 220) and the control group (*n* = 241) on the basis of the grouping criteria (Supplementary Table S1). Propensity score matching (PSM) was performed on a 1:1 scale on the basis of sex and age. In the final study analysis, 370 patients were divided into two groups: the ASCVD group, which included 185 patients with ASCVD (160 men; mean age 74.54 ± 12.15 years old), and the control group, which included 185 subjects without ASCVD (153 men; mean age 74.66 ± 11.91 years old). Among the ASCVD group, there were 161 cases of CAD, 1 case of ischemic stroke, 8 cases of peripheral artery stenosis, 9 cases of carotid artery stenosis, 4 cases of CAD combined with ischemic cerebrovascular disease, and 2 cases of carotid artery combined with peripheral artery stenosis.

The two groups had similar demographic characteristics, distributions of pubertal stages and anthropometric measurements (Table 1). However, there were notable differences in the levels of Cr (*P* = 0.006), total cholesterol (TC, *P* < 0.001), low-density lipoprotein cholesterol (LDL-c, *P* < 0.001), and LVEF (*P* = 0.035) between ASCVD patients and controls. ASCVD patients presented lower concentrations of TC and LDL-c, which may be associated with the use of lipid-lowering drugs.

**Table 1:** Baseline demographic characteristics of the ASCVD and control groups.

Characteristics	ASCVD (*n* = 185)	Control (*n* = 185)	*P*
Clinical parameters			
Male (*n*, %)	160 (86.49%)	153 (82.70%)	0.315
Age (years)	74.54 ± 12.15	74.66 ± 11.91	0.921
BMI (kg/m^2^)	24.81 ± 3.36	24.41 ± 2.94	0.221
SBP (mmHg)	135.55 ± 18.42	137.64 ± 15.90	0.243
DBP (mmHg)	73.22 ± 12.02	75.14 ± 10.07	0.097
Heart rate (bpm)	67.90 ± 11.69	67.79 ± 9.35	0.922
Medical history (*n*, %)			
Smoking history	74 (40.00%)	69 (37.30%)	0.595
Family history of early-onset cardiovascular disease	23 (12.43%)	23 (12.43%)	1.000
Hypertension	140 (75.68%)	143 (77.30%)	0.714
Dyslipidemia	182 (98.38%)	179 (96.80%)	<0.001**
Diabetes mellitus	95 (51.35%)	90 (48.65%)	0.604
Atrial fibrillation/flutter	16 (8.65%)	11 (5.95%)	0.526
Hyperuricemia	44 (23.78%)	40 (21.62%)	0.621
Cancer	22 (11.89%)	27 (14.59%)	0.445
Use of lipid-lowering drugs	182 (98.38%)	156 (84.32%)	<0.001**
Laboratory values			
ALT (IU/L)	25.97 ± 52.45	23.01 ± 19.82	0.474
AST (IU/L)	24.91 ± 21.40	24.89 ± 13.94	0.989
Creatinine (µmol/L)	94.01 ± 34.12	85.74 ± 22.00	0.006**
eGFR (ml/min/1.73 m^2^)	73.87 ± 53.57	74.39 ± 15.92	0.899
Uric acid (µmol/L)	348.97 ± 88.05	352.98 ± 78.53	0.645
Fasting blood-glucose (mmol/L)	6.47 ± 2.06	6.29 ± 2.07	0.392
Triglyceride (mmol/L)	1.50 ± 1.18	1.56 ± 0.98	0.544
TCHO (mmol/L)	3.62 ± 0.90	4.27 ± 1.03	<0.001**
HDL-c (mmol/L)	1.12 ± 0.30	1.17 ± 0.25	0.075
LDL-c (mmol/L)	1.94 ± 0.93	2.37 ± 0.85	<0.001**
Glycosylated hemoglobin (%)	6.74 ± 4.03	6.26 ± 1.01	0.117
LVEF (%)	63.56 ± 6.50	64.85 ± 5.21	0.035*

BMI, body mass index; SBP, systolic blood pressure; DBP, diastolic blood pressure; ALT, alanine aminotransferase; AST, aspartate aminotransferase; TCHO, total cholesterol; HDL-c, high density cholesterol; LDL-c, low density cholesterol; hs-CRP, hypersensitive C reactive protein; LVEF, left ventricular ejection fraction.

* *P* < 0.05.

** *P* < 0.01.

### 3.2 Plasma NRG-1 levels and their correlation with proinflammatory mediators

**Figure 2:**
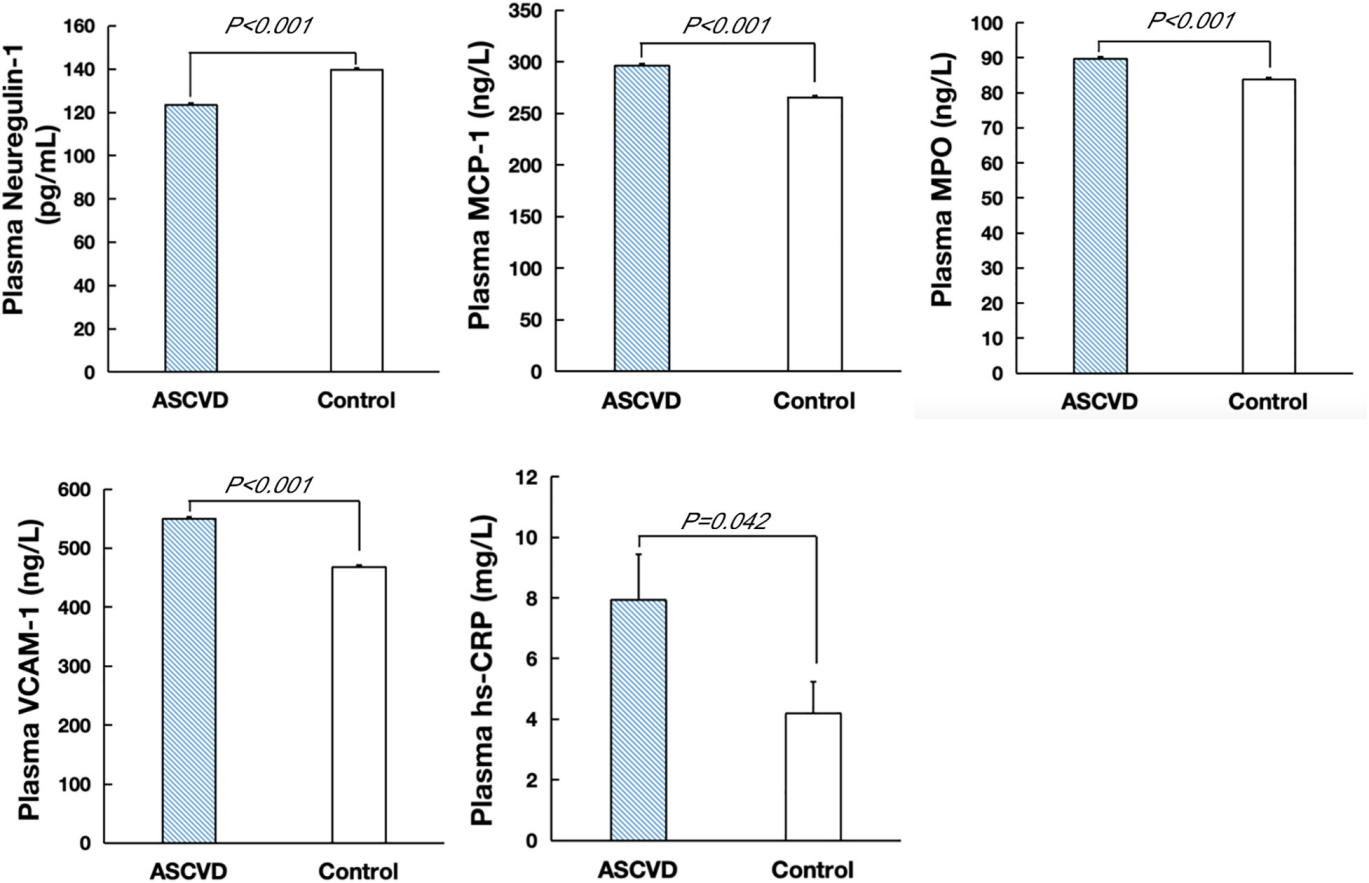
Plasma levels of NRG-1, MCP-1, MPO, VCAM-1 and hs-CRP in ASCVD and control groups.

NRG-1 levels were significantly lower in ASCVD patients than in the controls (123.45 ± 0.87 pg/ml vs. 139.76 ± 0.83 pg/ml, *P* < 0.001), whereas the concentrations of proinflammatory mediators such as MCP-1, MPO, VCAM-1, and hs-CRP were higher in ASCVD patients than in the controls (Figure 2, Supplementary Table S2).

Spearman's correlation analysis revealed that human circulating NRG-1 levels were negatively associated with MCP-1 (−0.278, *P* < 0.001), MPO (−0.171, *P* = 0.001) and VCAM-1 (−0.351, *P* < 0.001) levels. However, there was no significant correlation between NRG-1 and CRP (Supplementary Table S3).

### 3.3 Plasma NRG-1 levels are independent protective factors against ASCVD

Logistic regression was used to determine whether high levels of NRG-1 are independently protective against ASCVD development. After adjustment for other covariables (age, sex, BMI, blood pressure, heart rate, smoking status, family history of early-onset CVD, hypertension, dyslipidemia, diabetes, hyperuricemia, use of lipid-lowering drugs, glycosylated hemoglobin, creatinine, eGFR, uric acid, glucose, TG, TCHO, HDL-c, LDL-c, LVEF, MCP-1, MPO and VCAM-1), a binary analysis revealed that among the set of influencing variables for ASCVD, NRG-1 remained an independent protective factor (OR = 0.855, 95% CI = 0.814–0.899, *P* < 0.001; Table 2). However, the OR for TCHO is below 1, which could be linked to the fact that a significant portion (338/360, 93.89%) of the participants in our study were using lipid-lowering drugs. The use of lipid-lowering drugs conferred cardiovascular protection (OR = 0.051, 95% CI = 0.008–0.316, *P* < 0.001).

**Table 2:** Logistic regression analysis of the plasma levels of NGR-1 in ASCVD patients and controls.

Variable	*OR*	95% CI	*P*
SBP	1.031	1.002–1.060	0.033
TCHO	0.306	0.174–0.539	<0.001
Use of lipid-lowering drugs	0.051	0.008–0.316	0.001
MCP-1	1.040	1.017–1.063	0.001
MPO	1.136	1.058–1.219	<0.001
VCAM-1	1.032	1.020–1.043	<0.001
NRG-1	0.855	0.814–0.899	<0.001

SBP, systolic blood pressure; TCHO, total cholesterol, MCP-1, monocyte chemotactic protein-1; MPO, myeloperoxidase; VCAM-1, vascular cell adhesion molecule-1; Neuregulin-1, NRG-1.

### 3.4 Mediation analysis results

Given that the protective effect of NRG-1 may be associated with inflammatory factors, we conducted a mediation analysis to evaluate the proportional contributions of inflammatory factors (MCP-1, MPO, VCAM-1, and hs-CRP) to the associations of the NRG-1 with ASCVD risk. According to the results of the mediation analyses (Table 3), MCP-1, MPO, and VCAM-1 accounted for 20.2%, 8.8%, and 30.1%, respectively, of the associations of NRG-1 with ASCVD. However, there were no significant mediating effects observed for hs-CRP.

**Table 3:** Mediation analysis to evaluate whether proinflammatory mediators mediated the associations of the NRG-1 and ASCVD.

Exposure→mediator→outcome	Total effect	Direct effect (95% CI)	Mediation effect (95% CI)	*P*	Mediation proportion
NRG-1→MCP-1→ASCVD	−0.175	−0.14 (−0.17 to −0.10)	−0.035 (−0.05 to −0.02)	<0.001	20.2%
NRG-1→MPO→ASCVD	−0.143	−0.13 (−0.16 to −0.10)	−0.013 (−0.02 to −0.01)	<0.001	8.8%
NRG-1→VCAM-1→ASCVD	−0.20	−0.14 (−0.18–0.10)	−0.060 (−0.08 to −0.04)	<0.001	30.1%
NRG-1→hs-CRP→ASCVD	−0.1208	−0.12 (−0.15–0.10)	−0.0008	>0.05	0.66%

Adjust: sex, age, smoke history, family history of early-onset cardiovascular disease, hypertension, dyslipidemia, diabetes mellitus, atrial fibrillation/flutter, hyperuricemia, taking lipid-lowering drugs, creatinine, eGFR, left ventricular ejection fraction (LVEF). MCP-1, monocyte chemotactic protein-1; MPO, myeloperoxidase; VCAM-1, vascular cell adhesion molecule-1; NRG-1, Neuregulin-1.

### 3.5 Follow-up for ASCVD patients

The follow-up period ranged from 14 to 16 months (mean 13.8 ± 1.7 months), during which 33 patients experienced MACCEs. This included 8 patients who were hospitalized for HF, 22 patients with ACS, 1 patient with malignant arrhythmia, 1 patient with stroke, and 1 patient with sudden cardiac death. The initial baseline characteristics and biomarker levels of the patients with ASCVD are presented in Supplementary Table S4 according to the occurrence of MACCEs during follow-up. The results revealed that the mean NRG-1 concentration in patients with MACCEs was 112.04 ± 1.24 pg/ml, which was lower than that in patients without MACCEs (125.93 ± 0.90 pg/ml, *P* < 0.001). However, no significant intergroup differences were detected in MCP-1 (294.89 ± 4.19 ng/L vs. 296.63 ± 1.89 ng/L, *P* = 0.707), MPO (88.74 ± 1.14 ng/ml vs. 89.95 ± 0.54 ng/ml, *P* = 0.338) and VCAM-1 levels (553.34 ± 8.52 ng/ml vs. 548.85 ± 3.80 ng/ml, *P* = 0.633).

### 3.6 Prognostic value of NRG-1 in patients with ASCVD

**Figure 3:**
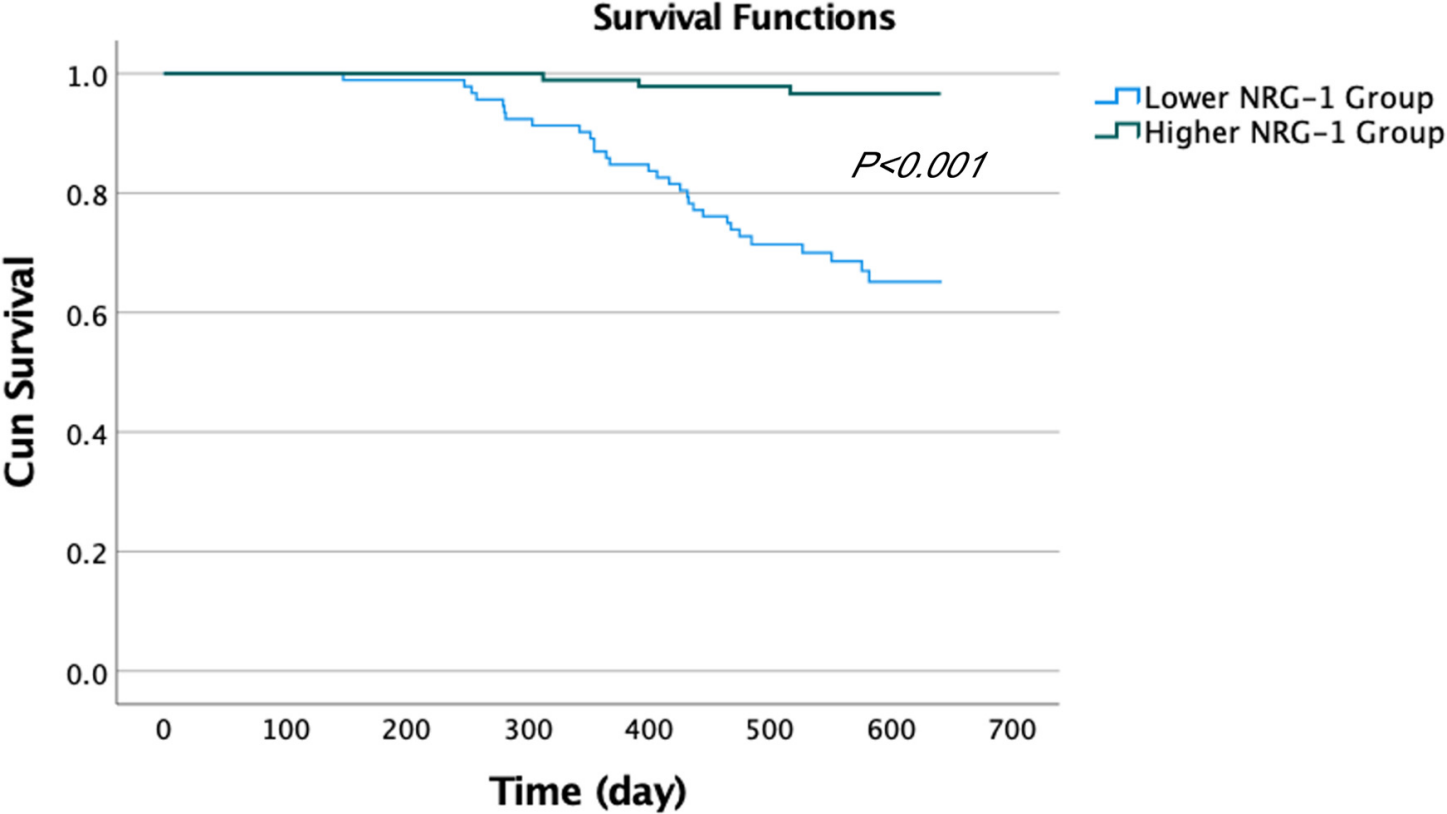
Kaplan–Meier survival curves according to different concentrations of NRG-1 (higher group: plasma NRG-1 levels ≥122.5 pg/ml; lower group: plasma NRG-1 levels <122.5 pg/ml). A log-rank test was used for the overall comparison among groups.

On the basis of the observed relationship between NRG-1 levels and the occurrence of MACCEs, we further evaluated the prognostic value of NRG-1 for predicting the occurrence of MACCEs. Patients with ASCVD were divided into two groups on the basis of their median NRG-1 level (122.5 pg/ml): the lower group (*n* = 92) and the higher group (*n* = 93). In the lower group (NRG-1 < 122.5 pg/ml), MACCEs were reported in 30 patients, which included 7 individuals hospitalized due to HF, 20 patients with ACS, 1 patient with severe arrhythmia, 1 patient with stroke, and 1 patient who experienced sudden cardiac death. In the higher group (NRG-1 ≥ 122.5 pg/ml), 3 patients were admitted to the hospital because of ACS recurrence or HF (Table 4). The Kaplan‒Meier survival curves suggested that patients with NRG-1 levels <122.5 pg/ml tended to have lower survival rates than did those with higher NRG-1 levels (Figure 3, *P* < 0.001).

**Table 4:** Occurrence of ASCVD patients with MACCEs in the lower and higher NRG-1 groups.

MACCEs (cases)	Lower NRG-1 group (<122.5 pg/ml, *n* = 92)	Higher NRG-1 group (≥122.5 pg/ml, *n* = 93)
Hospitalized due to HF	7	1
ACS	20	2
Severe arrhythmia	1	0
Stroke	1	0
Sudden cardiac death	1	0

MACCEs, major adverse cardiovascular and cerebrovascular events; ACS, acute coronary syndrome.

According to the cox models, the unadjusted hazard ratio was 0.892 (95% CI, 0.852–0.934). After adjusting for demographics, this association between NRG-1 and MACCEs remained significant (Table 5, Model 1). With the addition of covariates associated with NRG-1 from our cross-sectional analyses (Model 2), there were minimal changes in the relative hazard (HR 0.860, 0.816–0.907, *P* < 0.001). Furthermore, in the fully adjusted model (Model 3), which included additional potential confounders (LVEF and the levels of proinflammatory mediators), significance was maintained (HR 0.857, 0.809–0.908, *P* < 0.001). No single covariate changed the univariate HR between NRG-1 and MACCEs by more than 10%, and the backward elimination method did not reveal further evidence of confounding factors. Thus, circulating NRG-1 was an independent predictor of MACCEs in patients with ASCVD.

**Table 5:** The association between NRG-1 and MACCEs.

Model	Covariates	HR (95% CI)	*P*
Unadjusted (*n* = 185)	None	0.892 (0.852–0.934)	<0.001
Model 1 (*n* = 185)	Age, gender	0.869 (0.826–0.914)	<0.001
Model 2 (*n* = 184)	Model 1& Smoking history, Family history of early-onset cardiovascular disease, Hypertension, Dyslipidemia, Diabetes mellitus, Atrial fibrillation/flutter, Hyperuricemia	0.860 (0.816–0.907)	<0.001
Model 3 (*n* = 184)	Model 2& LVEF, MCP-1, MPO, VCAM-1, hs-CRP	0.857 (0.809–0.908)	<0.001

HR, hazard ratio; CI, confidence interval.

### 3.7 Subgroup analysis

**Figure 4:**
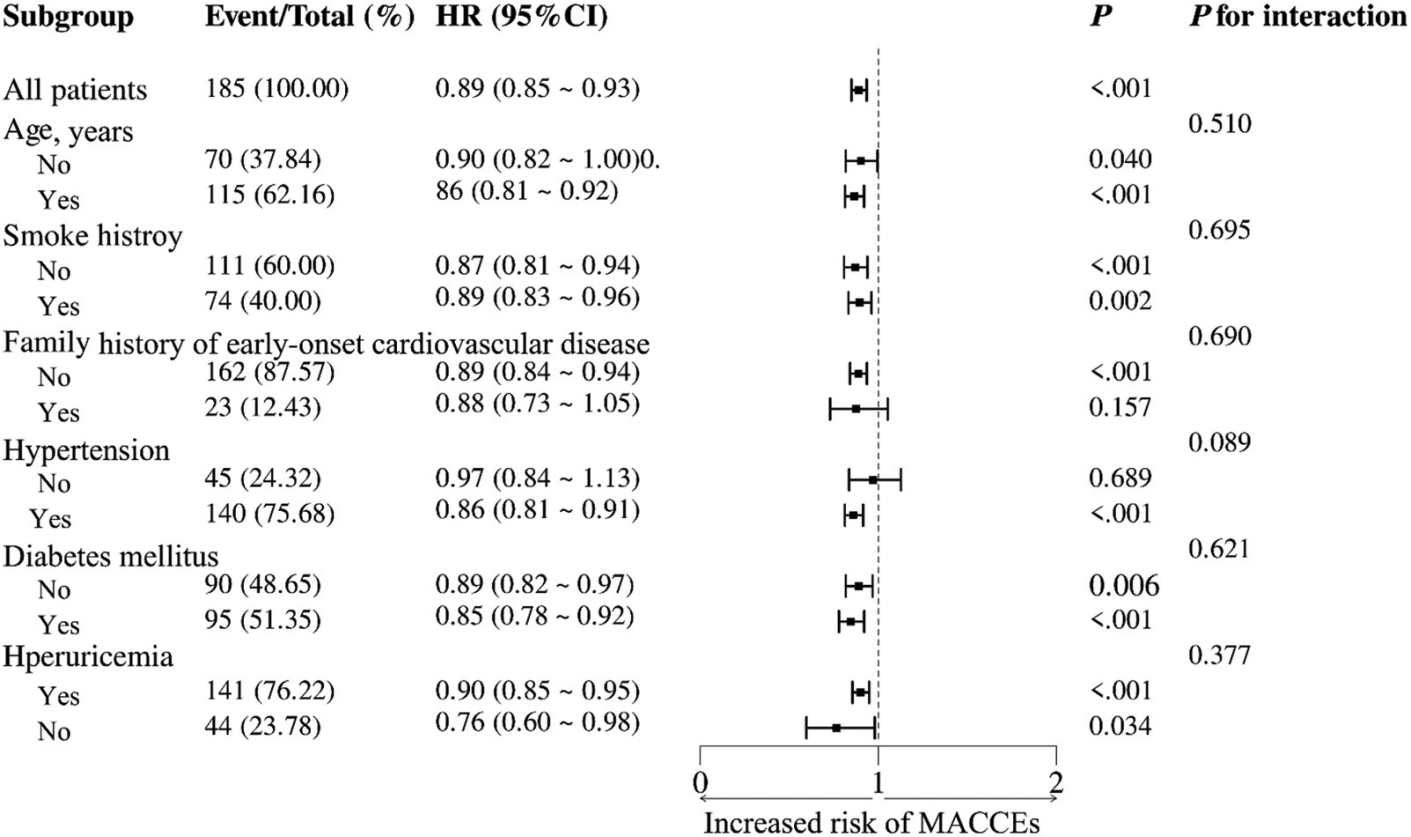
Subgroup analyses of the association between the NRG-1 level and the risk of MACCEs in patients with ASCVD.

To further investigate the relationship between the NRG-1 and MACCEs in patients with ASCVD, a series of subgroup analyses were conducted. As shown in Figure 4, none of the subgroups, including age, smoking history, family history of early-onset cardiovascular disease, and prevalence of hypertension, diabetes mellitus or hyperuricemia, profoundly changed the relationship between NRG-1 and MACCEs (all *P* values for interaction >0.05).

## 4 Discussion

NRG-1 is a member of the EGF family of proteins, is secreted and released by endothelial vascular cells, and affects the cardiovascular system. Many studies have shown that NRG-1 can repair the heart in the pathophysiology of atherosclerosis, MI, IR, HF, cardiomyopathy and other CVDs. Although it has been researched broadly in HF patients, NRG-1 has not been widely investigated in the context of ASCVD. Thus, in the present study, we investigated the levels of NRG-1 in ASCVD patients and their relationships with the levels of proinflammatory mediators and the prognosis of ASCVD patients. Our results revealed that plasma NRG-1 levels were significantly lower in patients with ASCVD than in controls and that reduced NRG-1 levels in ASCVD patients increased the risk of MACCEs. Thus, NRG-1 is a valuable laboratory indicator for the prognosis of patients with ASCVD.

Our findings fill knowledge gaps regarding the implications of lower plasma NRG-1 levels in patients with ASCVD. A previous study revealed that patients with acute coronary syndrome (ACS) or T2DM patients wtih high CAD risk have lower levels of NRG-1 than healthy controls or hypertensive controls. Circulating NRG-1 levels are inversely related to the severity of CAD lesions (27, 28). Haller et al. reported that NRG-1 plasma levels decreased significantly following percutaneous coronary intervention (PCI)/remote ischemic conditioning (RIC) and remained decreased up to 1 month following acute MI (29). The serum NRG-1 levels in T2DM patients with CVD risk and T2DM patients without CVD risk were significantly lower than those in healthy control subjects and were strongly predictive factor for increasing the risk of developing CVD in T2DM patients (30). Our study highlights the importance and difference of changes in circulating NRG-1 in ASCVD and HF patients. Ky Bonnie et al. reported that circulating NRG-1β was significantly elevated in patients with HF, with increasing levels according to disease severity (31). Heart failure may induce an increase in NRG-1β as a favorable compensatory response, similar to the effect of natriuretic peptides, and elevated NRG-1β reflects the underlying disease severity. Extensive work in animal models has demonstrated a cardioprotective effect of NRG-1. Thus, researchers propose that elevated NRG-1β is a compensatory factor, not a pathogenic factor, in the context of heart failure. Our results provide evidence that NRG-1 may be utilized as a potentially useful biomarker corresponding with cardiovascular and cerebrovascular ischemic stress. The decrease of NRG-1 may exacerbate oxidative stress-induced atherosclerotic plaque development, attributable to the loss of its cardioprotective anti-inflammatory and antioxidant functions.

Correlation analysis demonstrated that NRG-1 levels were negatively associated with MCP-1, MPO and VCAM-1 levels. The results of the mediation analysis revealed that MCP-1, MPO, and VCAM-1 levels had significant negative mediation effects on NRG-1 and ASCVD with percentages of 20.2%, 8.8%, and 30.1%, respectively. Elevated proinflammatory cytokine (e.g., TNF-α, MCP-1, and VCAM-1), ROS (32) and MPO (33) levels are associated with inflammatory status and increased oxidative stress. The possible mechanism involves NRG-1 suppressing ROS production by activating ERK1/2 inhibition of NADPH oxidase 4 (NOX4) activity and inhibiting the NLRP3/caspase-1 pathway, thereby attenuating myocardial oxidative damage and inflammatory responses (34). Additionally, NRG-1 exerts an antioxidant effect on myocardial tissue and endothelial cells through the activation of the AKT/eNOS pathway and diminishes the proinflammatory response by attenuating COX-2 expression in monocytes (35–37). In apolipoprotein E (ApoE) knockout mice, chronic administration of rNRG-1 suppressed the development of atherosclerotic lesions (24, 27). De Keulenaer et al. reported that rNRG-1 decreases inflammatory responses by diminishing the expression of MCP-1, interleukin-1β, VCAM-1, matrix metalloproteinase-9 (MMP-9), and cyclooxygenase-2 in monocytes (38). Thus, our results showed that a decrease in NRG-1 levels led to an increase in the levels of some proinflammatory cytokines, which aggravated the progression of ASCVD.

The follow-up data revealed that the average NRG-1 levels of patients who experienced MACCEs were lower than those of patients who did not, and the survival curves were significantly different. Therefore, decreased NRG-1 may signify an increased risk of MACCEs in ASCVD patients, and the underlying mechanism may involve the following aspects. First, the decrease in NRG-1 levels weakens its ability to suppress NOX4 activity through the activation of the ERK1/2 pathway, resulting in increased release of proinflammatory cytokines, which contributes to the progression of ASCVD. Second, NRG-1/ErbB activates several signaling pathways to protect against myocardial IR injury, which involves endoplasmic reticulum stress, calcium overload, oxidative stress and apoptosis (39). The main signaling pathways include the PI3K/Akt and Erk/MAPK pathways, which are both important reperfusion injury salvage kinases (40). The lower NRG-1 levels result in reduced activation of ErbB receptors and their downstream signaling pathways, such as the PI3K/Akt and Erk/MAPK pathways, which weakens the energy metabolism and antioxidant capacity of myocardial cells and leads to further deterioration of cardiac function. Moreover, activation of the NRG1-ErbB2 signaling pathway helps decrease glucose consumption after myocardial damage and promotes the utilization of glucose for energy (41), whereas a reduction in NRG-1 levels diminishes the protective effects of cardiac energy metabolism.

Circulating NRG-1 could serve as an independent predictor of short-term survival in patients with ASCVD. Cox regression demonstrated that none of the covariates significantly altered the HR of NRG-1 for MACCEs, indicating the robustness of NRG-1's prognostic value in this cohort. In the group with MACCEs, the reduction in NRG-1 levels was more pronounced than the reductions in the other inflammatory indicators, which may be related to the role of NRG-1 in antiatherosclerosis through multiple mechanisms. Previous studies have indicated that inflammatory biomarkers such as CRP (42), MPO (33), TNF-α (9), MCP-1 (43), and VCAM-1 (10) can predict the risk of future CAD, plaque stability, and adverse clinical outcomes in CAD patients. However, there were no differences in the levels of MCP-1, MPO, or VCAM-1 between the MACCE group and the non-MACCE group in our study. Our results were inconsistent with previous research findings, which may be related to various factors such as the study population, disease status, comorbidities, and small sample size. While no significant differences were observed in inflammatory factors between the two groups, the NRG-1 levels exhibited a marked discrepancy, indicating its potential as a superior predictor of MACCEs occurrence in ASCVD patients. Furthermore, we observed that none of the variables used to subgroup patients (including age, smoking history, family history of early-onset cardiovascular disease, and prevalence of hypertension, diabetes mellitus or hyperuricemia) had a significant effect on the relationship between the NRG-1 level and MACCEs, which highlights the applicability of our findings to the majority of individuals.

In summary, this is the first study to provide evidence that plasma NRG-1 concentrations are significantly reduced in patients with ASCVD and that measuring NRG-1 concentrations could provide a novel indicator for predicting the prognosis of ASCVD. On the basis of the cardioprotective effect of NRG-1, the development of drugs or therapeutic strategies targeting the NRG-1/ErbB signaling pathway may lead to innovative avenues for the treatment of ASCVD. This study has several limitations. First, it was a single-center study with a short follow-up duration and a relatively small sample size. Second, owing to the cross-sectional design of this study, it was not possible to establish a causal relationship between circulating NRG-1 levels and the risk of developing ASCVD. Additional large-scale prospective studies are needed to elucidate the exact relationship between NRG-1 levels and the risk of developing ASCVD.

## 5 Conclusion

Taken together, this is the first study to provide evidence that plasma NRG-1 concentrations are significantly reduced in patients with ASCVD. Reduced plasma NRG-1 levels in ASCVD patients increase the risk of MACCEs, and NRG-1 levels may serve as a useful laboratory marker for monitoring ASCVD prognosis. On the basis of the cardioprotective effect of NRG-1, the development of drugs or therapeutic strategies targeting the NRG-1/ErbB signaling pathway may lead to innovative avenues for the treatment of ASCVD.

## Data Availability

The raw data supporting the conclusions of this article will be made available by the authors, without undue reservation.
