# Supplementary material for: Reduced plasma levels of neuregulin-1 are associated with adverse outcomes in patients with atherosclerotic cardiovascular disease
Source: Front Cardiovasc Med. 2025 Jul 17;12:1600480. doi: 10.3389/fcvm.2025.1600480 (PMC12310740; doi:10.3389/fcvm.2025.1600480)
Supplement: Supplementary file 1 [file Datasheet1.docx]

Supplementary table 1. Baseline demographic characteristics of the ASCVD

and control groups before PSM

| Characteristics | ASCVD (n=220) | Control (n=241) | *P* |
| --- | --- | --- | --- |
| CLINICAL PARAMETERS |  |  |  |
| Male (n, %) | 188 (85.45%) | 193 (80.08%) | 0.127 |
| Age (years) | 76.94±12.66 | 71.49±12.40 | <0.001 |
| BMI (kg/m^2^) | 24.68±3.35 | 24.54±2.88 | 0.623 |
| SBP (mmHg) | 136.38±18.27 | 135.57±16.05 | 0.613 |
| DBP (mmHg) | 72.30±12.51 | 75.93±10.03 | 0.001 |
| Heart rate (bpm) | 67.55±12.31 | 68.17±9.705 | 0.546 |
| MEDICAL HISTORY (n, %) |  |  |  |
| Smoking history | 84 (38.18%) | 92(38.17%) | 0.999 |
| Family history of early-onset cardiovascular disease | 27 (12.27%) | 32(13.28%) | 0.748 |
| Hypertension | 171 (77.73%) | 171(70.95%) | 0.096 |
| Dyslipidemia | 216 (98.18%) | 232 (96.27%) | 0.208 |
| Diabetes mellitus | 111 (50.45%) | 119 (49.38%) | 0.818 |
| Atrial fibrillation/flutter | 22 (10.00%) | 12 (4.98%) | 0.041 |
| Hyperuricemia | 52 (23.64%) | 56 (23.24%) | 0.920 |
| Cancer | 31 (14.09%) | 27 (11.20%) | 0.352 |
| Use of lipid-lowering drugs | 216 (98.18%) | 232 (96.27%) | 0.208 |
| LABORATORY VALUES |  |  |  |
| ALT (IU/L) | 25.03±48.32 | 23.16±18.21 | 0.578 |
| AST (IU/L) | 24.50±19.94 | 24.29±12.55 | 0.885 |
| Creatinine (µmol/L) | 96.8±40.94 | 83.76±20.69 | <0.001** |
| eGFR (ml/min/1.73 m^2^) | 71.19±50.11 | 77.23±16.62 | 0.078 |
| Uric acid (µmol/L) | 346.42±90.61 | 351.65±76.35 | 0.502 |
| Fasting blood-glucose (mmol/L) | 6.51±2.10 | 6.22±1.90 | 0.114 |
| Triglyceride (mmol/L) | 1.48±1.11 | 1.58±1.02 | 0.325 |
| TCHO (mmol/L) | 3.60±0.89 | 4.48±2.59 | <0.001** |
| HDL-c (mmol/L) | 1.12±0.31 | 1.18±0.26 | 0.014 |
| LDL-c (mmol/L) | 1.92±0.88 | 2.42±0.86 | <0.001** |
| Glycosylated hemoglobin (%) | 6.70±3.72 | 6.21±0.95 | 0.057 |
| LVEF (%) | 63.44±6.53 | 65.14±5.28 | 0.002** |

**P*<0.05, ***P*<0.01; BMI, body mass index; SBP, systolic blood pressure; DBP, diastolic blood pressure; ALT, alanine aminotransferase; AST, aspartate aminotransferase; TCHO= total cholesterol; HDL-c, high density cholesterol; LDL-c, low density cholesterol; hs-CRP= hypersensitive C reactive protein; LVEF, left ventricular ejection fraction

| Characteristic | ASCVD（n=185） | Control（n=185） | *P* |
| --- | --- | --- | --- |
| NRG-1 (pg/mL) | 123.45±0.87 | 139.76±0.83 | <0.001 |
| MCP-1 (ng/L) | 296.32±1.71 | 265.25±1.80 | <0.001 |
| MPO (ng/L) | 89.74±0.48 | 83.74±0.52 | <0.001 |
| VCAM-1 (ng/L) | 549.66±3.46 | 468.13±3.58 | <0.001 |
| hs-CRP (mg/L) | 7.93±1.50 | 4.19±1.05 | 0.042* |

Supplementary table 2. Plasma levels of NRG-1and proinflammatory mediators in ASCVD patients and controls (mean± SE)

NRG-1= Neuregulin-1; MCP-1= monocyte chemotactic protein-1; MPO= myeloperoxidase; VCAM-1= vascular cell adhesion molecule-1; hs-CRP= hypersensitive C reactive protein

Supplementary table 3. NRG-1 is associated with proinflammatory mediators

in all subjects

| Variable | Correlation coefficient | *P* |
| --- | --- | --- |
| MCP-1 | -0.278 | <0.001 |
| MPO | -0.171 | 0.001 |
| VCAM-1 | -0.351 | <0.001 |
| hs-CRP | -0.055 | 0.291 |

MCP-1, monocyte chemotactic protein-1; MPO, myeloperoxidase; VCAM-1, vascular cell adhesion molecule-1

Supplementary table 4 Initial demographic characteristics of ASCVD patients,

stratified based on the occurrence of MACCEs during follow-up

| Characteristics | MACCEs (n=33) | Non-MACCEs (n=152) | *P* |
| --- | --- | --- | --- |
| CLINICAL PARAMETERS |  |  |  |
| Male (n, %) | 31 (93.94%) | 129 (84.87%) | 0.081 |
| Age（years） | 76.06±10.85 | 74.21±12.42 | 0.429 |
| BMI (kg/m^2^) | 24.66±3.49 | 24.85±3.34 | 0.769 |
| SBP (mmHg) | 130.30±16.17 | 136.68±18.73 | 0.071 |
| DBP (mmHg) | 70.45±11.53 | 67.35±11.69 | 0.380 |
| Heart rate (bpm) | 67.90±11.69 | 67.79±9.35 | 0.168 |
| MEDICAL HISTORY (n, %) |  |  |  |
| Smoke hisptory | 16 (48.48%) | 58 (38.16%) | 0.103 |
| Family history of early-onset cardiovascular disease | 4 (12.12%) | 19 (12.5%) | 0.953 |
| Hypertension | 28 (84.85%) | 112 (73.68%) | 0.131 |
| Dyslipidemia | 33（100%） | 149（98.03%） | 0.419 |
| Diabetes mellitus | 20 (60.61%) | 75 (49.34%) | 0.244 |
| Atrial fibrillation/flutter | 4 (12.12%) | 11 (7.24%) | 0.374 |
| Hyperuricemia | 6 (18.18%) | 38 (25.00%) | 0.407 |
| Use of lipid-lowering drugs | 33（100%） | 149（98.03%） | 0.419 |
| Cancer | 2 (6.06%) | 20 (13.16%) | 0.164 |
| LABORATORY VALUES |  |  |  |
| Hemoglobin (g/L) | 138.27±17.74 | 137.01±17.39 | 0.708 |
| ALT (IU/L) | 42.61±111.40 | 22.35±25.46 | 0.307 |
| AST (IU/L) | 24.21±9.66 | 25.07±23.20 | 0.836 |
| Creatinine (µmol/L) | 93.15±22.20 | 94.19±36.25 | 0.874 |
| eGFR (ml/min/1.73 m^2^) | 69.93±16.04 | 74.73±58.64 | 0.642 |
| Uric acid (µmol/L) | 340.15±83.19 | 350.89±89.23 | 0.527 |
| Fasting blood-glucose (mmol/L) | 6.34±1.52 | 6.50±2.16 | 0.697 |
| Triglyceride (mmol/L) | 1.42±0.94 | 1.51±1.23 | 0.685 |
| TCHO (mmol/L) | 3.44±0.92 | 3.65±0.89 | 0.210 |
| HDL-c (mmol/L) | 1.11±0.31 | 1.12±0.30 | 0.853 |
| LDL-c (mmol/L) | 2.10±1.68 | 1.90±0.66 | 0.502 |
| Glycosylated hemoglobin (%) | 6.60±1.41 | 6.77±4.40 | 0.828 |
| LVEF (%) | 64.15±5.10 | 63.43±6.78 | 0.562 |
| MCP-1 (ng/L) | 294.89±4.19 | 296.63±1.89 | 0.707 |
| MPO (ng/L) | 88.74±1.14 | 89.95±0.54 | 0.338 |
| VCAM-1 (ng/L) | 553.34±8.52 | 548.85±3.80 | 0.633 |
| NRG-1 (pg/mL) | 112.04±1.24 | 125.93±0.90 | <0.001** |

**P*<0.05, ***P*<0.01; BMI, body mass index; SBP, systolic blood pressure; DBP, diastolic blood pressure; ALT, alanine aminotransferase; AST, aspartate aminotransferase; TCHO= total cholesterol; HDL-c, high density cholesterol; LDL-c, low density cholesterol; LVEF, left ventricular ejection fraction; MCP-1, monocyte chemotactic protein-1; MPO, myeloperoxidase; VCAM-1, vascular cell adhesion molecule-1; NRG-1, Neuregulin-1
